# High Boron-loaded DNA-Oligomers as Potential Boron Neutron Capture Therapy and Antisense Oligonucleotide Dual-Action Anticancer Agents ^‡^

**DOI:** 10.3390/molecules22091393

**Published:** 2017-08-23

**Authors:** Damian Kaniowski, Katarzyna Ebenryter-Olbińska, Milena Sobczak, Błażej Wojtczak, Sławomir Janczak, Zbigniew J. Leśnikowski, Barbara Nawrot

**Affiliations:** 1 Centre of Molecular and Macromolecular Studies, Polish Academy of Sciences, Department of Bioorganic Chemistry, Sienkiewicza 112, 90-363 Lodz, Poland; dkanio@cbmm.lodz.pl (D.K.); kebenryt@cbmm.lodz.pl (K.E.-O.); milena@cbmm.lodz.pl (M.S.); 2 Institute of Medical Biology of the Polish Academy of Sciences, Laboratory of Molecular Virology and Biological Chemistry, 106 Lodowa St., 92-232 Lodz, Poland; b.wojtczak@biogeo.uw.edu.pl (B.W.); sjanczak@cbm.pan.pl (S.J.)

**Keywords:** metallacarborane, antisense oligonucleotide, BNCT, ROS, EGFR

## Abstract

Boron cluster-modified therapeutic nucleic acids with improved properties are of interest in gene therapy and in cancer boron neutron capture therapy (BNCT). High metallacarborane-loaded antisense oligonucleotides (ASOs) targeting epidermal growth factor receptor (EGFR) were synthesized through post-synthetic Cu (I)-assisted “click” conjugation of alkyne-modified DNA-oligonucleotides with a boron cluster alkyl azide component. The obtained oligomers exhibited increased lipophilicity compared to their non-modified precursors, while their binding affinity to complementary DNA and RNA strands was slightly decreased. Multiple metallacarborane residues present in the oligonucleotide chain, each containing 18 B-H groups, enabled the use of IR spectroscopy as a convenient analytical method for these oligomers based on the diagnostic B-H signal at 2400–2650 cm^−1^. The silencing activity of boron cluster-modified ASOs used at higher concentrations was similar to that of unmodified oligonucleotides. The screened ASOs, when used in low concentrations (up to 50 μM), exhibited pro-oxidative properties by inducing ROS production and an increase in mitochondrial activities in HeLa cells. In contrast, when used at higher concentrations, the ASOs exhibited anti-oxidative properties by lowering ROS species levels. In the HeLa cells (tested in the MTT assay) treated (without lipofectamine) or transfected with the screened compounds, the mitochondrial activity remained equal to the control level or only slightly changed (±30%). These findings may be useful in the design of dual-action boron cluster-modified therapeutic nucleic acids with combined antisense and anti-oxidant properties.

## 1. Introduction

Boron neutron capture therapy (BNCT) is a therapeutic modality for the treatment of cancers based on the absorption of low-energy neutrons by nonradioactive boron-10 (^10^B) atoms delivered to neoplastic cells in the form of a boron-carrying drug [1,2]. For BNCT to be successful, ca. 10^9^ atoms of ^10^B must be localized on or preferably within the neoplastic cells (i.e., 20–40 μg of B in one gram of tumor), and a sufficient number of thermal neutrons must be delivered to sustain a lethal ^10^B (n, alpha) lithium-7 reaction. Therefore, the development of high boron-loaded, tumor-selective drugs will play an important role for BNCT to evolve into a clinically accepted cancer treatment. A number of potential boron carriers have been synthesized and tested, though only two, l-4-(dihydroxyboryl) phenylalanine (BPA) and the sodium salt of thioborane anion (Na_2_B_12_H_11_SH, BSH), are in clinical use [3,4].

To date fission reactors have been used as a neutron source for BNCT [5]. This has been one of the major obstacles limiting the application of BNCT as a broadly applied anticancer therapy. Emerging accelerator-based sources of epithermal neutrons, which may be placed on-site in hospitals, may revolutionize the future use of BNCT [6,7]. A simultaneous increase in the demand for new, more efficient methods to develop boron carriers can also be expected. In this report, we describe a new approach for designing boron carriers based on nucleic acid structures. Though the idea was proposed by us earlier [8], the method allowing for the loading of nucleic acids with multiple boron cluster groups was only recently developed [9]. A 22-mer antisense oligodeoxyribonucleotide targeting mRNA of epidermal growth factor receptor (EGFR) was chosen as a nucleic acid template model [10,11]. Use of other methods such as targeted liposomal delivery systems may increase the selectivity of boronated oligonucleotides transport into cancer cells.

The EGFR gene is overexpressed in several human cancers and is considered a reasonable anticancer therapy target. Other nucleic acid sequences that target other cancer-related genes can be used depending upon the specific needs. However, activation of the EGFR signaling pathway in cancer cells has been linked not only with increased cell proliferation but also with angiogenesis, metastasis, and decreased apoptosis. Additionally, preclinical data have confirmed that many anti-EGFR agents have the potential to increase the effectiveness of current cytotoxic drugs [12]. Thus, the rationale for EGFR-targeted approaches to cancer treatment is apparent and now well-established. The development of agents with binary activities, such as boron carriers for BNCT and anti-EGFR activities, represents a novel approach to cancer treatment.

In this communication, we describe the synthesis of high boron cluster-loaded antisense oligonucleotides (ASOs) targeting EGFR and describe their physicochemical properties—lipophilicity, binding affinity to complementary DNA and RNA strands, circular dichroism (CD) and infrared (IR) spectroscopic features, as well as their biological potential-silencing, cytotoxic, and pro-and anti-oxidant activities.

## 2. Results and Discussion

### 2.1. Chemistry: Synthesis of Boron Cluster Conjugated Oligonucleotides

**Scheme 1:**
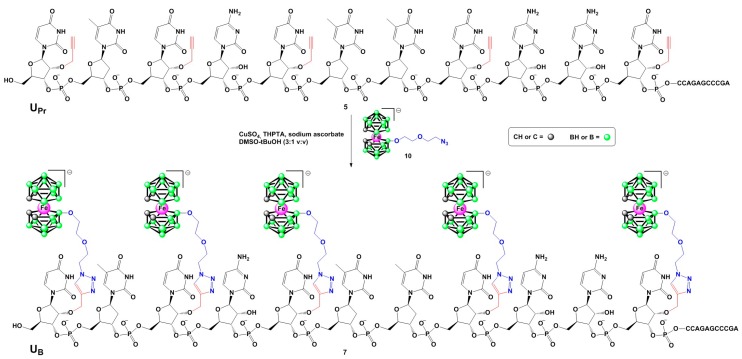
Synthesis of oligonucleotide **7** bearing five boron cluster units via copper-catalyzed azide-alkyne cycloaddition of alkyne derivative **5** with metallacarborane-alkyl azide **10**. Oligonucleotide **6** was obtained analogously from its alkyne precursor **4**.

Synthesis of boron cluster-modified anti-EGFR antisense oligonucleotides **6** and **7** (Table 1) was performed in two steps (Scheme 1) according to a recently developed general approach allowing for the incorporation of various types of boron clusters into specific locations of oligonucleotide chains [9]. First, boron cluster acceptor oligonucleotides bearing 2′-*O*-propargyluridine (U_Pr_) units, which are derivatives of the parent non-modified antisense oligonucleotide **1**, were synthesized. They were obtained in two variants, with four or five thymidine units replaced with U_Pr_, giving oligonucleotides **4** and **5**, respectively (Table 1). The alkyne-functionalized DNA oligonucleotides and the non-modified DNA and RNA complementary oligonucleotides (**2** and **3**) were synthesized according to a standard phosphoramidite solid-phase method. A long chain alkyl amine controlled pore glass (LCA CPG) support and commercially available nucleoside phosphoramidites with phenoxyacetyl (Pac) protecting groups removable under mild alkaline conditions were utilized. Synthesis of the oligonucleotides at the 0.1 µmole scale was performed on an automated DNA synthesizer under conditions recommended by the manufacturer. All the compounds were cleaved from the solid support as 5′-4,4′-dimethoxytrityl-(DMT)-derivatives and purified by reversed-phase high- performance liquid chromatography (RP-HPLC) (“DMT-on” approach [13]). The deprotection of the 5′-OH groups was performed on C18 SepPak cartridges with 2% TFA (trifluoroacetic acid). The sequence and purity of the obtained oligonucleotides were confirmed by matrix assisted laser desorption ionization-time of flight mass spectrometry (MALDI-TOF MS) and analytical RP-HPLC (Table 1).

**Figure 1:**
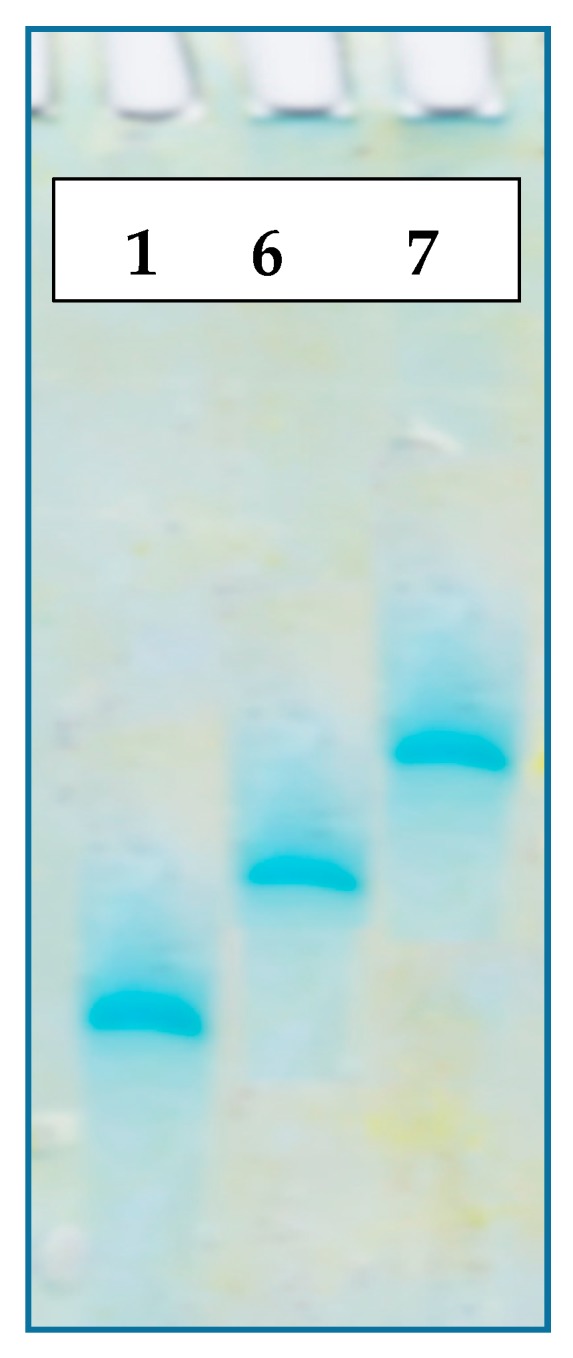
Electrophoretic analysis of the non-modified reference oligonucleotide **1** and the boron cluster-modified DNA oligonucleotides **6** and **7**. All of the oligomers are 22-nt long and contain 0, 4 or 5 modified U_B_ units.

In the second step, the alkyne-functionalized oligonucleotides **4** and **5** were post-synthetically conjugated with an alkyl azide boron cluster donor containing a negatively charged [(3,3′-iron-1,2,1′,2′-dicarbollide)(−1)]ate (**10**, Scheme 1) using a standard copper-catalyzed Huisgen-Meldal-Sharpless azide-alkyne cycloaddition (click reaction) [14,15,16]. The final products were isolated by RP-HPLC and their molecular mass and purity were confirmed by MALDI-TOF mass spectrometry (Table 1) and polyacrylamide/7 M urea gel electrophoresis (PAGE) (Figure 1).

A decreased PAGE mobility of the oligonucleotides **6** and **7** in comparison to their parent non-modified oligomer **1** is observed, because of the increased molecular mass of the modified oligomers, which is not balanced by their increased total negative charge.

### 2.2. Physicochemical Characterization of Oligomers ***6*** and ***7***

#### 2.2.1. Lipophilicity of the Oligonucleotide Conjugates with Boron Clusters Measured as Affinity towards the Reversed Phase HPLC Column

Due to properties of the boron cluster-modifying unit, oligonucleotides modified with boron clusters are known for their high affinity to lipophilic reversed phase (C18) HPLC columns [17]. Here, we compared the chromatographic mobility of oligonucleotides **8** and **9** containing one or two boron cluster units U_B_ located at positions 7 or 7 and 11, respectively [9], with those for oligonucleotides **6** and **7** with four or five U_B_ units, respectively.

**Figure 2:**
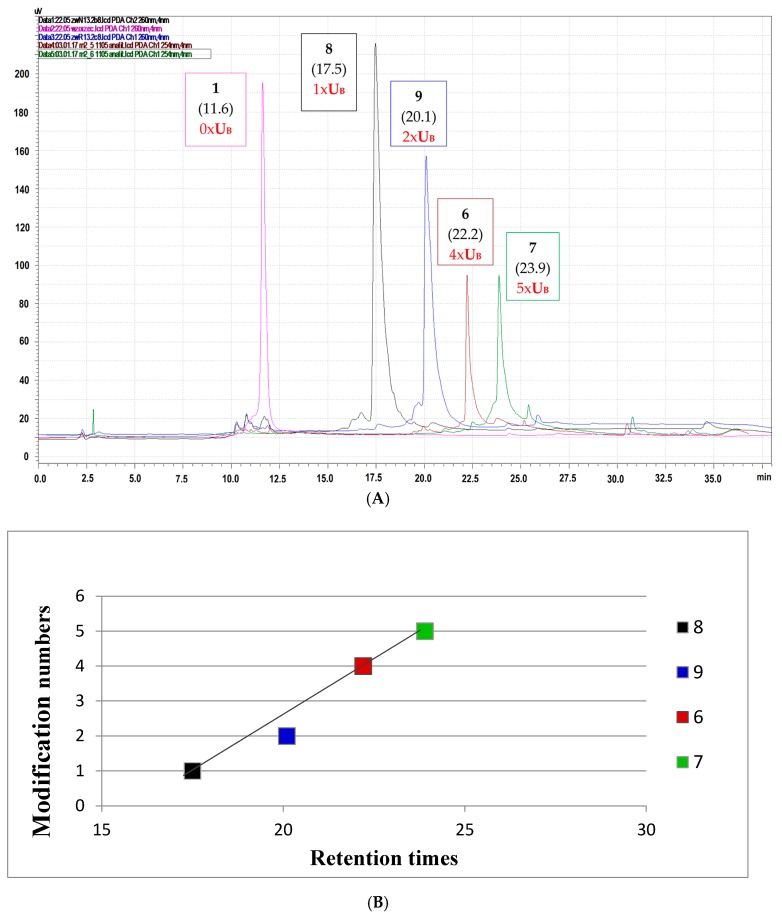
(**A**) RP-HPLC profiles of oligonucleotides **6** and **7**, which contain four or five boron cluster units, respectively, and oligomers **8** and **9** with one or two modifications, respectively [9], compared to the mobility of the non-modified reference oligomer **1**; (**B**) Boron cluster content-dependent chromatographic mobility reflecting oligomer lipophilicity. Retention times (in min.) are given in brackets.

For these measurements, a highly hydrophobic elution buffer system, in which buffer A was 0.1 M ammonium acetate and buffer B was 100% acetonitrile, was used. As shown in Figure 2A, the chromatographic mobility of the test oligomers increased with the increasing U_B_ unit contents in the analyzed oligomer (Table 1, Figure 2A), and this dependence was almost linear with respect to the number of negatively charged [(3,3′-iron-1,2,1′,2′-dicarbollide)(−1)]ate residues (Figure 2B). Our trials to determine the partition coefficient with the standard shake-flask water/1-octanol method failed in this case, probably due to the surfactant-like properties of oligonucleotides **6** and **7**.

#### 2.2.2. Circular Dichroism (CD) Measurements of Boron Cluster-Conjugated Oligomers **6** and **7** with Their DNA and RNA Complementary Strands (**2** and **3**, Respectively)

**Figure 3:**
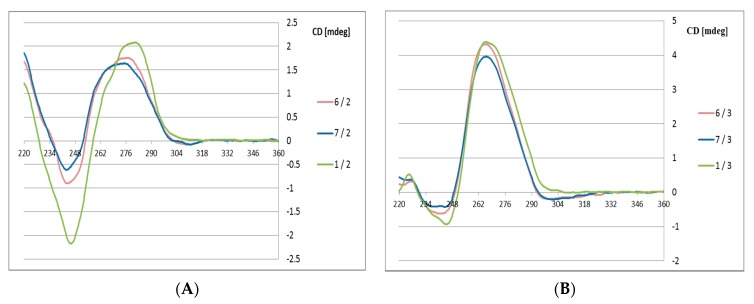
Circular dichroism spectra for the **6/2**, **7/2** and **1/2** duplexes with a DNA complementary strand (**A**) and for the **6/3**, **7/3** and **1/3** duplexes with an RNA complementary strand (**B**).

The high boron cluster-modified oligomers **6** and **7** were annealed with their complementary DNA or RNA strands **2** and **3**, respectively, in phosphate buffer (pH 7.4). The CD curves of the **6**/**2** and **7**/**2** duplexes (Figure 3) exhibit some deviations from the curve observed for the reference duplex **1**/**2** with the B-type helical structure.

Variations are observed at the Cotton effect intensities at 280 nm (positive band) and at 248 nm (negative band), which suggests that the duplexes with DNA template exhibit lower helicity in the presence of the structurally constrained boron cluster substituents. In contrast, duplexes **6** and **7** with RNA templates (**6**/**3** and **7**/**3**) adopt an A-type helical structures (Figure 3B), and only minute deviations are observed compared to the reference **1**/**3** duplex. This observation may be of interest for possible applications of these antisense oligonucleotides, which should form stable duplexes with target mRNA strands

#### 2.2.3. Thermodynamic Properties of Duplexes of High Boron Cluster-Modified Antisense Oligomers **6** and **7** with DNA and RNA Templates

**Figure 4:**
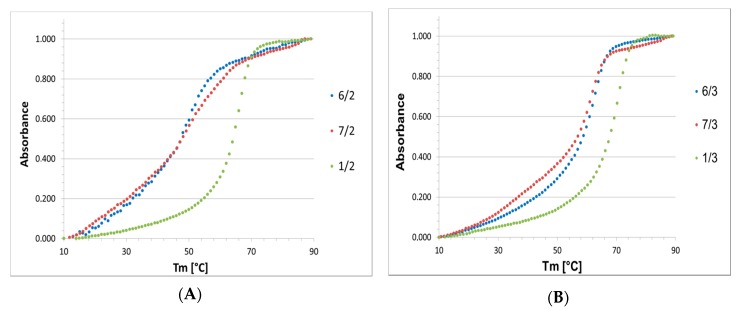
UV-monitored, normalized thermal dissociation curves for the **6/2**, **7/2** and **1/2** duplexes with a DNA complementary strand (**A**) and the **6/3**, **7/3** and **1/3** duplexes with an RNA complementary strand (**B**).

High boron cluster-modified oligomers **6** and **7**, which annealed with complementary DNA or RNA strands **2** and **3**, respectively, were submitted to UV-monitored thermal dissociation at temperatures ranging from 10 to 90 °C. The obtained melting profiles (Figure 4) allowed us to use a two-state model to calculate the thermodynamic parameters, including the enthalpy, entropy and Gibbs free energy, of the DNA/DNA and DNA/RNA duplex transitions to single strands with the MeltWin program (the Meltwin software license was kindly provided by Jeffrey McDowell, www.meltwin.com).

**Figure 5:**
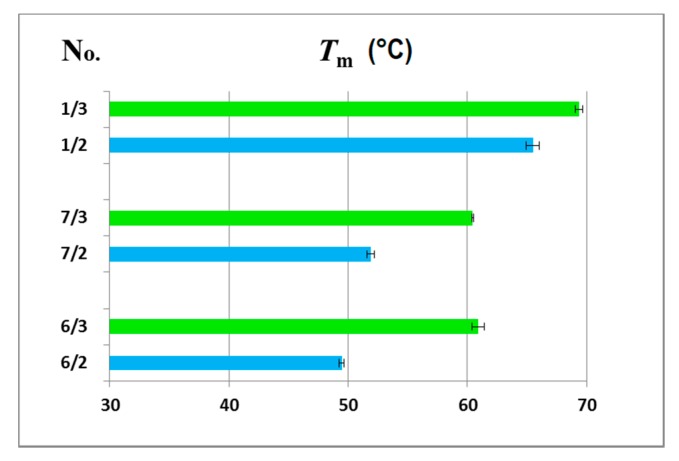
Comparison of the melting temperatures (*T*_m_ (°C)) for the **6/2**, **7/2** and **1/2** duplexes with a DNA complementary strand (blue bars) and the **6/3**, **7/3** and **1/3** duplexes with an RNA template (green bars).

The thermodynamic data for the melting transitions and melting temperatures are shown in Table 2, and the graphical representation of the melting temperatures is presented in Figure 5. The measurements have shown that ASO/RNA duplexes were thermodynamically much more stable than their ASO/DNA counterparts, with ΔG differences up to 4 kcal/mol and Δ*T*_m_ near 10 °C, while this difference was only 4 °C for reference duplexes **1/2** and **1/3**. 

The observed differences are most likely due to structural features of the C3′-*endo* sugar-linked boron cluster units U_B_, which facilitate formation of A-type U_B_-DNA **6** and **7** duplexes with an RNA template. This structural property of DNA/RNA duplexes was also observed in the CD spectrum for the **1/3** reference duplex, demonstrating helical structures and the adoption of an A-type helical mode (Figure 3B). In contrast, boron cluster unit U_B_-containing ASOs form less stable B-type ASO/DNA helices. The CD spectra also indicate some distortions in the **6**/**2** and **7**/**2** duplexes from a typical B-type structure. The reasonable thermodynamic stability of the boron cluster-ASO/RNA duplexes with the U_B_-type-modified units is advantageous for the application of these modified ASOs for the recognition of their target mRNA sequences and duplex formation, which are prerequisites for gene expression regulation via the antisense approach.

#### 2.2.4. Infrared Spectroscopy Analysis

**Figure 6:**
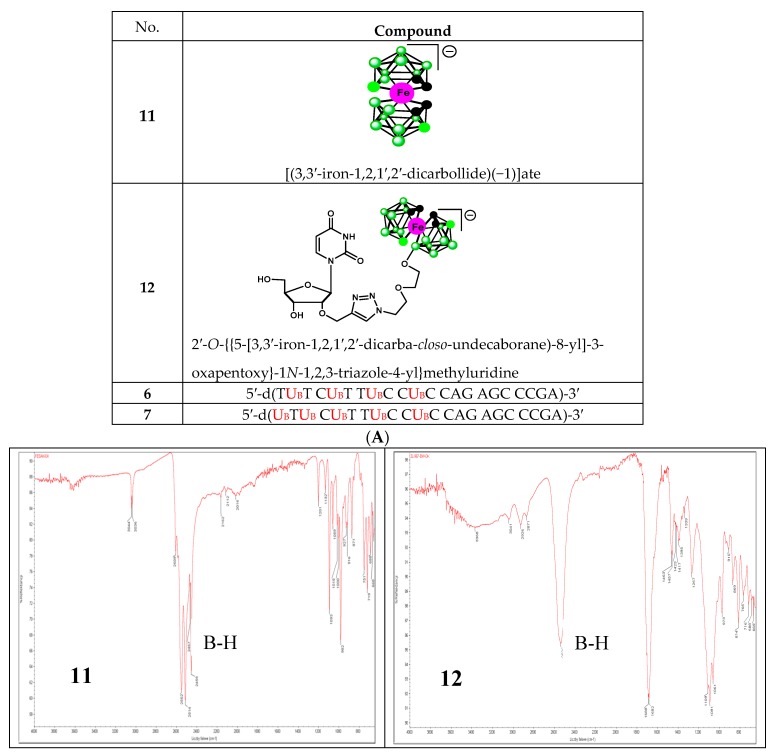

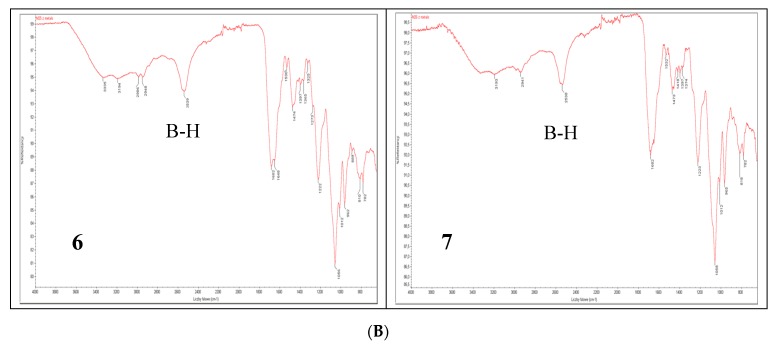
Structures of the oligonucleotides containing four and five metallacarborane residues, **6** and **7**, respectively, and their simple metallacarborane models, which include free metallacarborane **11** and its 2′-*O*-uridine derivative **12** (**A**, modified units U_PR_ and U_B_ highlighted in red) and their infrared spectra (**B**). B-H bands at 2400–2650 cm^−1^ region are marked.

To date, infrared light sensitive markers and infrared spectroscopy have rarely been used as a detection method for nucleic acids. This is mainly due to the lack of labels that absorb in the 1900–2600 cm^−1^ window where nucleic acids (and other organic molecules) are transparent. Boron cluster-modified DNA or RNA oligonucleotides offer a unique opportunity in this respect [18]. The B-H stretch of these compounds (**6** and **7**) occurs in the 2400–2650 cm^−1^ region of the IR spectrum (Figure 6B lower panel). For comparison, the IR spectra of metallacarborane **11** and its 2′-*O*-uridine derivative **12** [19] (see the structures in Figure 6A) are also shown (Figure 6B upper panel). Extended fragments of the IR spectra of **6**,**7**,**11** and **12** in the region of 2800–2200 cm^−1^ are shown at Figure S2C. An application of IR for the detection of an oligonucleotide probe with a single carborane (C_2_B_10_H_12_) or metallacarborane [[(3,3′-cobalt-1,2,1′,2′-dicarbollide)(−1)]ate; 3,3′-Co-(1,2-C_2_B_9_H11)_2_]^−^ modification has been previously demonstrated using a B-H vibration as a diagnostic signal [18]. Herein, we show the further development of this infrared labeling approach based on a “click chemistry” method that allows for the incorporation of multiple metallacarborane labels, each containing 18 B-H groups, which enables a substantial strengthening of the diagnostic B-H signal (Figure 6B, lower panel) and an increase in the sensitivity of detection.

### 2.3. Biological Properties of High Boron Cluster-Loaded Antisense Oligonucleotides ***6*** and ***7***

#### 2.3.1. Silencing Activity of Boron Cluster-Modified Anti-EGFR Antisense Oligonucleotides **6** and **7**

**Figure 7:**
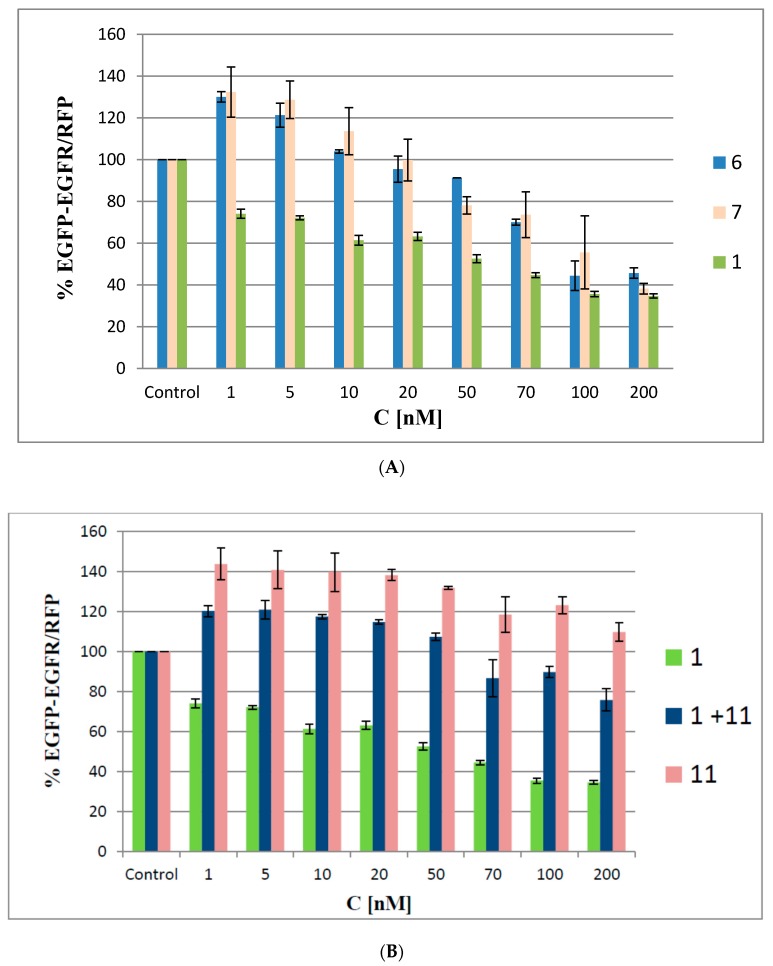
Concentration-dependent silencing activities of boron cluster-modified oligonucleotides **6** and **7** (**A**) and of a non-modified antisense oligonucleotide **1**, metallacarborane **11**, and their mixture (**1** + **11**) (**B**), as determined by a pEGFP-EGFR/RFP dual fluorescence assay in HeLa cells. The cells were transfected with the pEGFP-EGFR and pDsRED-N1 plasmids and then treated (in the presence of Lipofectamine) with the respective oligonucleotide at concentrations ranging from 1–200 nM. Metallacarborane **11** was added to the cells after the oligonucleotide transfection was finished and the medium was exchanged. The cells were incubated for next 48 h. The relative EGFP-EGFR/RFP fluorescence of the cells transfected with the plasmids only was assessed as 100%. The results are mean values from three independent experiments.

We used a dual (green and red) fluorescence assay (DFA) to analyze the antisense activities of oligonucleotides **6** and **7**. This reporting system is based on monitoring of the fluorescence levels of green fluorescent protein (EGFP) and red fluorescent protein (RFP), both of which are expressed in HeLa cells from the exogenously delivered pEGFP-C1 and pDsRed2-N1 bacterial plasmids, respectively. In our earlier work, we described the construction of the pEGFP-EGFR fusion plasmid, in which the gene of interest (in our case, a 330-nt fragment of the EGFR gene) was fused to the EGFP gene [9]. Here, the cells were co-transfected with the pEGFP-EGFR and pDsRED-N1 plasmids, and then treated with oligonucleotides **6** and **7** or with the non-modified oligonucleotide **1** with concentrations ranging from 1 nM to 200 nM. The level of relative EGFP-EGFR/RFP fluorescence of the transfected cells was compared to the fluorescence of the cells expressing both plasmids but not transfected with the ASOs (48 h assay). As shown in Figure 7A, the antisense activity of the reference ASO **1** is concentration-dependent and resulted in a linear decrease in EGFR protein levels down to 40% of the control at 100 nM. In contrast, when oligomers **6** and **7** were applied, the EGFP-EGFR fusion protein fluorescence levels were initially elevated to approximately 130%. This effect was observed for minute amounts (1–5 nM) of ASOs **6** and **7** only. When the tested ASOs were used at higher concentrations (>50 nM), a gradual decrease in target protein expression levels (down to 40% of the control at 200 nM) was observed.

We hypothesize that the increased expression of EGFP-EGFR observed at the beginning of the assay may represent a possible cellular defense process against the ASO boron cluster components that stimulates cell metabolism and leads to increased expression of the transfected plasmids. The defense process may occur due to the generation of ROS by the metallacarborane unit bearing a redox-active iron ion component [20]. Using oligonucleotides **6** and **7** at higher concentrations (20–70 nM) led to a gradual decrease in EGFP-EGFR protein expression, though **6** and **7** were still less active than the non-modified reference ASO. This result suggests that the boron cluster pendants affect the recognition of the RNA in the DNA/RNA duplex by RNase H, the cleavage-executing ribonuclease, and inhibit its endonucleolytic activity. The highest silencing activity of oligomers **6** and **7** was observed at concentrations above 100 nM, where all the tested ASOs exhibited similar silencing activities (~60%).

To support the above hypothesis that the increased expression of EGFP-EGFR may originate from a possible cellular defense against the ASOs’ boron cluster components, we screened an effect of metallacarborane **11** on the EGFR-GFP expression under the same conditions as above. As shown in Figure 7B, metallacarborane alone, given in the 5-fold higher concentration corresponding to that for **7** stimulates the cell metabolism and increases the expression of the GFP-EGRF protein up to >140%, compared to the control level. At concentrations above 50 nM (corresponding to 250 nM of **11**) decreased fluorescence is observed, probably due to some toxicity. However, in the cells transfected with **1** and then supplemented with **11**, over the entire concentration range the GFP-EGFR expression was higher than that for **1** alone, but ca. 20–35% lower than for metallacarborane **11** alone. We suggest that the presence of **11** (at the concentration 5 times higher than **1**) and its 48 h presence in the cell medium induces low formation of ROS, which stimulate cell metabolism and lead to the increased expression of the exogenous plasmids. The observed effect is more pronounced than that observed for the oligonucleotides **6** and **7** bearing metallacarborane moieties (transfected to the cells over 5 h only). In contrast, neither a control non-sense oligonucleotide **13**, nor its mixture with metallacarborane **11** exhibited remarkable antisense activity (Figure S3). Therefore, we conclude, that the observed effects of GFP-EGFR expression upon transfection with the metallacarborane conjugates **6** and **7** originates mostly from the antisense properties of ASOs.

#### 2.3.2. Measurement of Reactive Oxygen Species (ROS) Generation by Metallacarborane, [(3,3′-Iron-1,2,1′,2′-dicarbollide)(−1)]ate Derivatives

Experiments with cells transfected with oligonucleotides **6** and **7**, which contain four and five metallacarborane units, respectively, were performed to study of the effect of the redox-active metal ion (Fe(III)) present in the [(3,3′-iron-1,2,1′,2′-dicarbollide)(−1)]ate U_B_ on the generation of reactive oxygen species (ROS) in HeLa cells. Cells transfected with non-modified reference oligonucleotide **1** were used as a control. Intracellular ROS levels were assessed with a 2,7-dichlorofluorescein diacetate (DCF-DA) dye assay. DCF-DA is a membrane-permeable reagent that diffuses across lipid membranes and, upon deacetylation, is subsequently converted into DCF, which is a membrane-impermeable form. Non-fluorescent DCF-DA is oxidized by intracellular ROS and forms highly fluorescent DCF. Thus, in the DCF-DA assay, the HeLa cells were transfected with oligonucleotides **6** or **7** or control oligonucleotide **1** at concentrations ranging from 1–200 nM.

**Figure 8:**
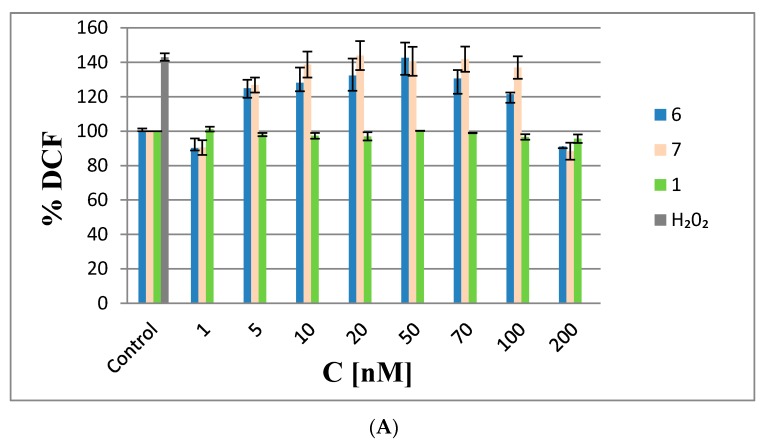

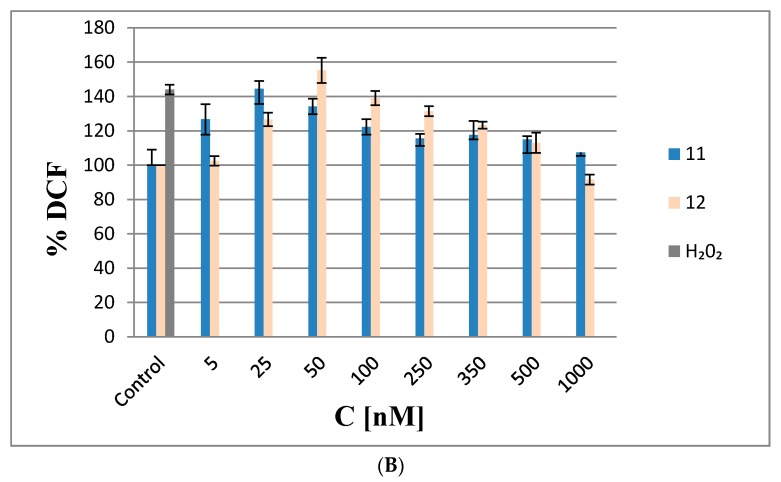
Analysis of ROS generation in HeLa cells. (**A**) Cells transfected with increasing amounts of boron cluster-containing oligonucleotides **6** and **7** or with non-modified oligonucleotide **1** (1–200 nM, 48 h) in the presence of Lipofectamine 2000; (**B**) HeLa cells treated with **11** and **12** (5–1000 nM). The non-transfected cells and cells treated with 1000 nM H_2_O_2_ (marked as H_2_O_2_) were used as controls. The results are mean values from three independent experiments.

As shown in Figure 8A, ROS levels in the HeLa cells transfected with **6** and **7** in the range from 5 to 20 nM increased by 25 and 50%, respectively, relative to the negative controls (non-transfected cells), indicating the pro-oxidative properties of the test ASOs at these low concentrations. The increased ROS levels remained constant up to 70 nM with **6** and **7**; at higher concentrations (above 70 nM), ROS levels decreased to the levels in the controls. The more visible effect at higher concentrations might be due to the anti-oxidative activities of the metallacarboranes, suggesting that **6** and **7** exhibit scavenging activities towards ROS species (similar to iron species involved in the redox cycle) [21]. Interestingly, non-modified oligonucleotide **1** did not generate ROS. Thus, it could be concluded that the observed effects are due the metallacarborane iron bis(1,2-dicarbollide)]ate present in **6** and **7**.

To verify this hypothesis, we performed ROS generation tests with metallacarborane (3,3′-iron bis(1,2,1′,2′-dicarbollide)]ate (**11**)) and its nucleoside conjugate 2′-*O*-{{5-[3,3′-iron-1,2,1′,2′-dicarba-*closo*-undecaborane)-8-yl]-3-oxapentoxy}-1*N*-1,2,3-triazole-4-yl}methyluridine (**12**) (see Figure 6A for structures). The HeLa cells were treated with **11** or **12** at five-fold higher molar concentrations than the oligonucleotides because 1 nM of **7** corresponds to 5 nM of **11** or **12** in terms of metallacarborane contents. The results presented in Figure 8B demonstrate a similar mode of ROS generation by compounds **11** and **12** as by oligonucleotides **6** and **7**, with similar concentration-dependent pro- and anti-oxidative properties. However, the lower molecular weight compounds **11** and **12** generate ROS more efficiently then the high molecular weight oligonucleotides **6** and **7**. This suggests higher ROS generation by the metallacarborane residue alone or when tethered to a low molecular weight component (150% ROS level at 25 nM of **11**) than when bound to the nucleic acid backbone (125% ROS level at 5 nM of **7**). Alternatively, more efficient cellular uptake for low molecular weight compounds **11** and **12** than for oligonucleotide **7**, could be responsible for the effects.

ASOs with metallacarboranes (**6** and **7**) containing redox-active Fe (III) ions that are delivered to cells generate ROS as a result of oxidative stress. At low concentrations, they stimulate the cells to elevate their metabolic activity. As the concentration of the supplied compounds increases, the amount of Fe (III) ions that scavenges free radicals also increases; thus revealing the anti-oxidative properties of these ASOs. When ASOs were used at the highest concentration (200 nM) in our experiments, the ROS levels were the same as in the control cells, suggesting that perhaps equilibrium between the rate of ROS formation and their scavenging is reached. Therefore, we assume that the silencing of EGFR by higher concentrations of oligonucleotide-metallocarborane conjugates will occur without increases in ROS in cancer BNCT therapy. It is worth noting that the control antisense and non-sense oligonucleotides, **1** and **13**, respectively, do not increase the ROS level significantly (ca. 10% increase of the DCF level by oligonucleotide **13**, Figure S4). Moreover, we also tested the effect of ferrocene, a ROS-inducing agent containing a Fe (II) ion, on the ROS level in HeLa cells transfected with the oligonucleotide **1**. Ferrocene used at micromolar concentrations is toxic for the cells (by the Fenton reaction, and HO^●^ radical generation) [22]. In the ROS experiments we used this agent at much lower concentrations (nanomolar), and we could not see any expected ROS inducing effect. Instead, as shown in Figure S3, ferrocene given as supplement of oligonucleotide **1**, at the concentration ≥250 nM (5-times higher compared to **1**) occurred toxic for the cells.

There are reports in the literature on drug/ROS/metastasis correlations that are related to the following two dependencies: (1) high doses of a drug are effective and (2) low doses, when inadequately administered and below the therapeutic dose, cause high oxidative stress resulting in cellular irritability in new tissues [23]. The discovery of the anti-oxidative properties of oligonucleotide-metallacarboranes at higher concentrations opens new possibilities for the development of new metallacarborane-based ROS scavenging agents that are simultaneously effective inhibitors of EGFR gene expression.

#### 2.3.3. Metabolic Activity of HeLa Cells Treated with Metallacarborane Derivatives

To determine whether the metallacarborane-containing ASOs can alter mitochondrial activities in cells, we screened compounds **6** and **7** in HeLa cells via the MTT assay. The MTT assay is a colorimetric assay for assessing cell metabolic activity, in which the positively charged small molecule [(3-(4,5-dimethylthiazol-2-yl)-2,5-diphenyltetrazolium bromide) tetrazolium] enters a cell and undergoes mitochondrial reductase/NADPH-mediated conversion to purple formazan. While the exact mechanism of MTT metabolism is unclear, it is evident that in living cells the mitochondria need to be intact and functioning to promote a positive MTT output [24].

**Figure 9:**
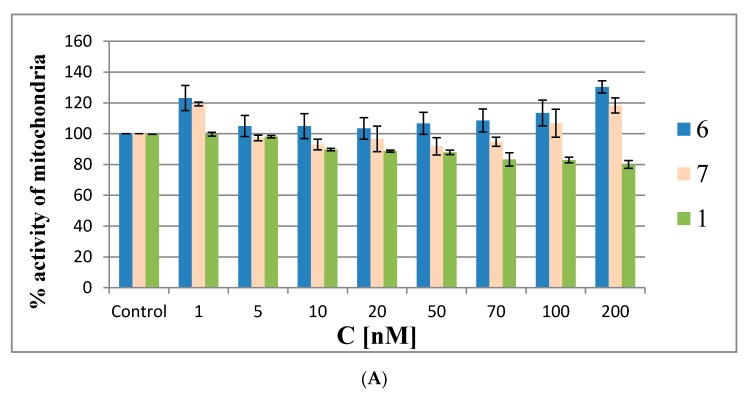

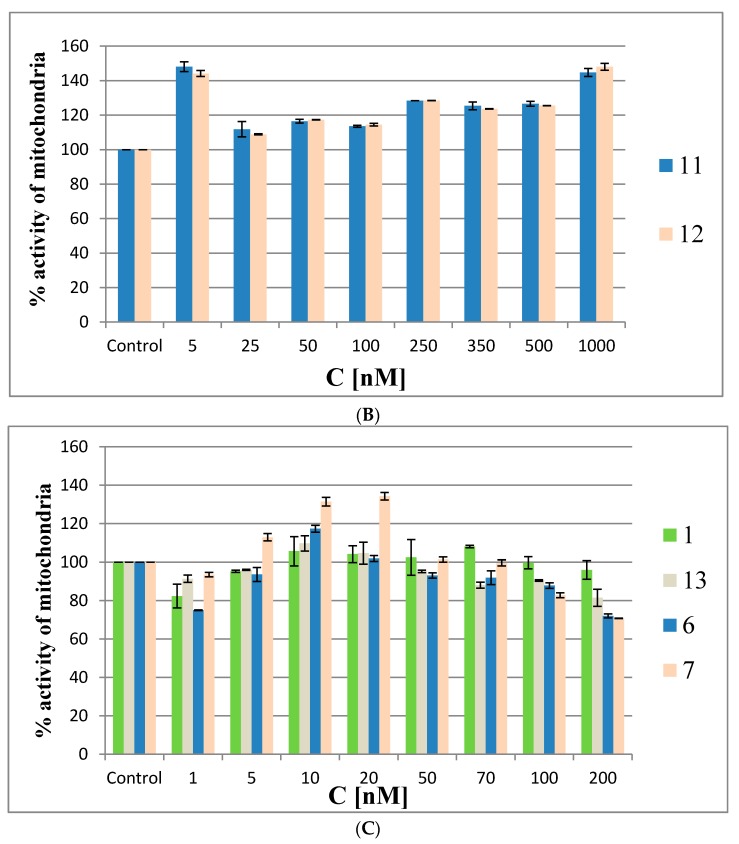
Mitochondrial activity by MTT assay in HeLa cells treated with metallacarborane derivatives **6** and **7** (**A**), **11** and **12** (B) and the reference oligonucleotides **1** and **13** compared to modified ASOs **6** and **7** (**C**) for 48 h.

The MTT assay was performed in HeLa cells with oligonucleotides **6** and **7** and reference **1**, as well as with the building block model compounds **11** and **12** at the same concentrations as described above for the ROS generation assay (48 h). In the first experiments (the results are shown in Figure 9A,B) the Lipofectamine 2000 transfection agent was omitted to avoid interference from possible cytotoxic effects due to the silencing of cellular EGFR by the antisense oligonucleotides. The MMT assay results are shown in Figure 9A for oligonucleotides **6**, **7** and **1** and in Figure 9B for compounds **11** and **12**. The MTT assays showed that the metabolic activity of HeLa cells treated with low concentrations of metallacarborane derivatives (1 or 5 nM) was increased by ~20% by oligonucleotides **6** and **7** and by ~50% by the building blocks **11** and **12**, respectively.

At the intermediate concentrations, the metabolic activity drops to the control level (20–50 nM for **6** and **7** and at 25 nM for **11** and **12**), and then gradually increases with increasing compound concentrations to 120–140% of the metabolic activity of the control cells. Interestingly, the MTT assay for the non-modified reference oligomer **1** demonstrated a slight cytotoxic effect (20%) towards the HeLa cells. The increased metabolic activity of the HeLa cells treated with low concentrations of metallacarborane derivatives **6**, **7**, **11** and **12** may result from the cells’ defense systems being activated against ROS generated in the presence of the Fe (III)-redox reactive agent [25,26]. This acceleration in cell metabolism at low concentrations of **6** and **7** agrees with the observed higher pEGFP-EGFR protein expression levels in the dual fluorescence assay at low ASO concentrations (1–10 nM) (Figure 7). The pro-oxidant properties of the Fe (III)-boron clusters are also demonstrated by the increased levels of ROS species in the HeLa cells when the ASOs are at low (nanomolar) concentrations (up to 20 nM, Figure 8). To clarify the mitochondrial activity of HeLa cells transfected with ASOs, we also performed the MTT assay with oligonucleotides **6**, **7**, antisense reference **1** and the non-sense oligonucleotide **13** [27] transfected to the cells with lipofectamine. The results shown in Figure 9C demonstrate that the mitochondrial activity of the cells only slightly decreased upon the highest concentrations of oligonucleotides (up to 30% for **6** and **7**), but in general, the test compounds are not cytotoxic for the HeLa cells. The assay reflects the ROS-inducing character of **6** and **7**, rather than their antisense activity against endogenous EGFR.

Remarkably, oligonucleotides **6** and **7**, when transfected with the assistance of Lipofectamine 2000, operate inside the cells and exhibit reasonable antisense silencing activities towards exogenously delivered the EGFR gene (up to 60%) compared to the control (Figure 7). They exhibit also anti-oxidant activities (Figure 8). However, in the MTT assay, where no Lipofectamine 2000 is used, these highly decorated polyanionic DNA strands probably cross the cellular membranes to a limited extent and adsorb rather at the cell surface. These interactions might induce ROS stress and enhance cell metabolism (in our case observed at the level of ~120–130% of the control, Figure 9A). Interestingly, among ROS-generating species, hydrogen peroxide activates numerous cellular pathways involved in cellular growth, survival, proliferation and metabolism and angiogenesis [28].

## 3. Materials and Methods

### 3.1 General Information

Chemicals were obtained from Aldrich Chemical Company (St. Louis, MO, USA) and were used without further purification unless otherwise stated. Unmodified nucleoside phosphoramidites and 5′-dimethoxytrityl-2′-propargyluridine 3′-*O*-(*N*,*N*-diisopropyl-2-cyanoethyl) phosphoramidite were purchased from ChemGenes Corporation (Wilmington, MA, USA). [(3,3′-Iron-1,2,1′,2′-dicarbollide)(−1)]ate cesium salt was bought from Katchem (Režn/Prague, Czech Republic). Flash chromatography was performed on silica gel 60 (230–400 mesh, Aldrich Chemical Company). R*_f_* values refer to analytical thin layer chromatography (TLC) performed using pre-coated silica gel 60 F_254_ plates purchased from Sigma-Aldrich (Steinheim, Germany) and developed in the solvent system indicated. Compounds were visualized with UV light (at λ_max_ = 254 nm). The yields are not optimized.

### 3.2. Mass Spectrometry and Ultraviolet Spectroscopy Measurements (UV)

The MALDI-TOF mass spectra were recorded on a Voyager Elite mass spectrometer (PerSeptive Biosystems Inc., Framingham, MA, USA) in the linear and negative ion modes. All UV-absorption measurements were performed in 1 cm^−1^ path length cells using a GBC Cintra 4040 UV-VIS spectrophotometer (Braeside, VIC, Australia). UV experiment samples for each compound were dissolved in deionized water, and the measurements were performed at ambient temperature.

### 3.3. Attenuated Total Reflectance−Fourier Transform Infrared (ATR−FTIR) Spectroscopy Measurements

Infrared absorption spectra were recorded using a Smart iTR diamond attenuated total reflectance (ATR) attachment on a Nicolet 6700 FT-IR spectrometer (Thermo Scientific, Waltham, MA, USA) equipped with an ETC EverGlo* source for the IR range, a Ge-on-KBr beam splitter, and a DLaTGS detector. Samples to be analyzed were placed on a diamond ATR element in solid form. The Omnic 8.1 software program (Thermo Fisher Scientific, Waltham, MA, USA) was used for data acquisition and processing.

### 3.4. Synthesis and Purification of DNA Oligonucleotides

The alkyne-functionalized DNA oligonucleotides **4** and **5** and non-modified DNA and RNA oligonucleotides **1**, **2** and **3** were synthesized according to the phosphoramidite solid-phase approach [29] using an LCA CPG support and commercially available nucleoside phosphoramidites with “mild” Pac protecting groups (ChemGenes). Synthesis of the oligonucleotides at the 0.1 μmole scale was performed on a Gene World DNA synthesizer (K&A, Schaafhein, Germany) under the conditions recommended by the manufacturer. All the compounds were cleaved from the solid support as 5′-DMT-derivatives and then purified by RP-HPLC according to a standard procedure. The proper sequence and the purity of the compounds were confirmed by MALDI-TOF mass spectrometry (Table 1). The syntheses of oligomers **1**–**3** furnished ca. 20–30 optical units (A_260_, OD) (>50%).

### 3.5. Synthesis and Purification of DNA Oligonucleotides Modified with Boron Cluster

The oligonucleotides **4** and **5** were post-synthetically modified with boron clusters containing negatively charged [(3,3′-iron-1,2,1′,2′-dicarbollide)(−1)]ate (**11**, Figure 6A) using a copper-catalyzed azide-alkyne cycloaddition reaction. Reactions were performed according to the standard copper sulfate procedure described in Glen Reports [30]. First, the following solutions were prepared: (1) DMSO (dimethylsulfoxide)/tBuOH (*t*-butanol) (3:1 *v*/*v*), (2) 0.1 M copper sulfate in water, (3) 0.2 M THPTA (tris (3-hydroxypropyltriazolylmethyl) amine) in DMSO/tBuOH and (4) 20 mM azide 10 in DMSO/tBuOH. Then, THPTA and copper sulfate solutions were mixed at a 1:1 ratio and degassed with an argon stream. Fifty molar equivalents of alkyl azide boron cluster **10** (0.897 μL) and 25 molar equivalents of the cooper sulfate/THPTA solution (2.4 μL) were added to a vial containing oligonucleotide **4** or **5** (2 OD) in water (37.48 μL). The mixture was vortexed and approximately 30 μL of DMSO/tBuOH (3:1, *v*/*v*) was added if a precipitate formed. Afterwards, 40 equivalents of freshly prepared 0.1 M sodium ascorbate in water (3.76 μL) were added to the reaction mixture. The final solution was briefly degassed with argon and agitated at room temperature for 4 h. Then, the products were isolated by RP-HPLC using a Kinetex 5 µm, C18, 250 × 4.6 mm column with buffer A (0.1 M CH_3_COONH_4_/H_2_O) and buffer B (100% CH_3_CN) at a 1 mL/min flow rate. The buffer gradient was as follows: (1) 0–2 min 0% B; (2) 2–25 min 0–70% B; (3) 25–30 min 70% B; (4) 30–35 min 70%–0% B; and (5) 35–38 min 0% B. UV detection was performed at λ_max_ = 268 nm. The collected oligonucleotide fractions were desalted on C18 SepPak cartridges (Waters Corp., Milford, MA, USA). The molecular mass of all the synthesized oligonucleotides was confirmed by MALDI-TOF mass spectrometry (Table 1) and their purity by polyacrylamide gel electrophoresis (PAGE) analysis (Figure 1). The oligomers **6** and **7** were obtained in 40–50% yield.

### 3.6. Electrophoretic Analysis Migration of ASOs in Polyacrylamide Gels

The electrophoretic analysis (PAGE) was run in 15% polyacrylamide gels containing 7 M urea at room temperature at a constant voltage of 300 V/cm and 6 mA/gel. Suitable separations were obtained in 3 h. The gel was stained in Stains-all for 30 min and scanned using a G-Box (Syngene, Cambridge, UK).

### 3.7. Melting Profiles and Thermodynamic Calculations

Absorbance measurements were performed in 1 cm^−1^ path length cells using a GBC Cintra 4040 UV-VIS spectrophotometer equipped with a Peltier Thermocell (Dandenong). The **6**/**2**, **7**/**2**, **1**/**2**, **6**/**3**, **7**/**3**, and **1**/**3** duplexes were dissolved in 10 mM Tris-HCl buffer (pH 7.4) containing 100 mM NaCl and 0.1 mM EDTA (disodium ethylenediaminetetraacetic acid) to final concentration of 1 μM. The melting profiles were recorded in the range from 10–90 °C with a temperature gradient of 1 °C/min. The melting temperatures were calculated using the first derivative method. Thermodynamic parameters (ΔG, ΔH and ΔS) were determined by numerical fitting of a given melting curve using a two-state model algorithm provided by the MeltWin v.3.5 software (Jeffrey McDowell, www.meltwin.com). Each result was taken as an average of three independent experiments.

### 3.8. Chromatographic Analysis of Lipophilicity for Oligonucleotides ***1*** and ***6***–***9***

HPLC analyses of the oligonucleotides modified with boron clusters (**6**–**9**) and the non-modified oligonucleotide (1, 0.2 OD) were performed on a Prominence HPLC system (Shimadzu, Kyoto, Japan) using a Kinetex C-18 5 µm (250 × 4.6 mm column, Phenomenex, Torrance, CA, USA). All of the analyses were performed at ambient temperature. The HPLC analysis used buffer A (0.1 M CH_3_COONH_4_) and buffer B (100% CH_3_CN) at a 1 mL/min flow rate. The buffer gradient was as follows: (1) 0–2 min 0% B; (2) 2–25 min 0–70% B; (3) 25–30 min 70% B; (4) 30–35 min 70%–0% B; and (5) 35–38 min 0% B. UV detection was performed at λ = 268 nm.

### 3.9. Circular Dichroism Measurements

CD measurements of the DNA and RNA duplexes were performed under a contractual service agreement with the Institute of Organic Chemistry, Polish Academy of Sciences in Warsaw. The spectra were recorded on a J-815 CD spectrometer (Jasco, Tokyo, Japan) at room temperature in the same buffer as for the melting experiments described above. The duplex concentration was 1 µM in a quartz cuvette with a 0.5 cm^−1^ path length and a 1 mL capacity. The following parameters were used for the CD: (1) 1.0 nm bandwidth, (2) 40 nm/min scan speed, (3) 0.5 s response time, and (4) 0.2 nm step resolution.

### 3.10. Cell line and Cell Culture Conditions

HeLa (human cervical carcinoma, ATCC, Manassas, VA, USA) cells were cultured in RPMI 1640 medium (Gibco BRL, Paisley, UK) supplemented with 10% heat-inactivated fetal bovine serum (FBS) (Gibco BRL), 100 U/mL penicillin, and 100 μg/mL streptomycin (Polfa, Tarchomin, Poland) at 37 °C and 5% CO_2_. Right before transfection, the culture medium was replaced and the cells were trypsinized and counted. Twenty-four hours before the experiment, the cells were plated in a 96-well plate (plates with black walls and a transparent bottom, Perkin-Elmer, Waltham, MA, USA) at a density of 15 × 10^3^ cells per well in a volume of 100 µL of full medium. On the day of transfection, the cells should be 80% confluent. Directly before the transfection, the cell medium containing antibiotics was replaced with 100 µL per well of antibiotics-free medium. The transfection was executed using the Lipofectamine 2000 transfection reagent (Invitrogen, New York, NY, USA) at a 2:1 ratio (2 μL of Lipofectamine 2000 per 1 μg of nucleic acid **6**, **7** or **1**) according to the manufacturer’s protocol. For the dual fluorescence assay (DFA), the HeLa cells were transfected with the reporter plasmid pDsRed-N1 (BD Biosciences, Franklin Lakes, NJ, USA) (45 ng/well), pEGFP-EGFR plasmid (100 ng/well) and suitable oligonucleotides with metallacarboranyl groups (1–200 nM) dissolved in 50 µL of OPTI-MEM medium (Gibco BRL). After 5 hours of incubation, the transfection mixture was replaced with 200 µL of fresh medium with antibiotics per well. For experiments with metallacarborane **11**, this compound was dissolved in DMSO and added to the cell medium at the concentration 5–1000 nM. Similar procedure was used for treatment of the cells with ferrocene. After 48 h of incubation at 37 °C in a 5% CO_2_ atmosphere, the cells were washed two times with PBS buffer (without Ca^2+^ and Mg^2+^) and lysed with NP-40 buffer (150 mM NaCl, 1% IGEPAL, 50 mM Tris-HCl pH 7.0, and 1 mM PMSF) overnight at 37 °C. The prepared cell lysates were used for fluorescence determination.

### 3.11. Fluorescence Measurements of Silencing Activity of ASOs

The pEGFP-EGFR (green fluorescent protein) and RFP (red fluorescent protein) fluorescence values were determined using a Synergy HT reader (BIO-TEK, Waltham, MA, USA). Quantification of the data was performed with the KC4 software (version 2.7, BIO-TEK). Excitation and emission wavelengths for each protein were as follows: (1) EGFP, λ_Ex_ = 485/20 nm and λ_Em_ = 528/20 nm and (2) RFP, λ_Ex_ = 530/25 nm and λ_Em_ = 590/30 nm. The antisense activities of the oligonucleotides were calculated as the ratio of EGFP-EGFR to RFP proteins fluorescence values according to the following equation: activity of oligonucleotides (%) = 100% − (sample EGFP_f_/RFP:control EGFP_f_/RFP) × 100%, where EGFP_f_ means the fluorescence value of the EGFP-EGFR fusion protein. Each average fluorescence value was the mean of eight repeats calculated after eliminating the extreme values. Each oligonucleotide activity value given on the plots is the average of mean values from three independent experiments. The fluorescence level (EGFP-EGFR/RFP) in the control cells (transfected with pDsRed-N1 and pEGFP-EGFR plasmids) was taken as a reference (100%).

### 3.12. Metabolic Activity of HeLa Cells Treated with Metallacarboranyl-Derivatives

The metabolic activities of oligonucleotides **1**, **6** and **7** as well as the model metallacarborane **11** and its derivative **12** was measured in HeLa cells using the MTT assay. The cells were plated at a density of 8000 cells per well (oligonucleotides concentrations ranging from 1–200 nM) in 96-well plates. The cells were incubated for 48 h at 37 °C in 5% CO_2_ and then MTT solution in PBS (5 mg/mL) was added to each well. Then, the cells were incubated for 3 h at 37 °C and 5% CO_2_. Finally, 95 μL of lysis buffer (NP-40, 20% SDS (sodium dodecyl sulfate), and 50% aqueous DMF (dimethylformamide), pH 4.5) was added to each well and incubated overnight at 37 °C. The plate absorbance was measured at two wavelengths, 570 nm and reference 630 nm (plate reader, colorless walls, Perkin-Elmer). The results are mean values from three independent experiments.

### 3.13. Intracellular ROS Measurements in Living Cell

The cells were plated at a density of 15,000 cells per well (oligonucleotides concentrations ranging from 1–200 nM) in 96-well plates. Directly before the transfection, the cell medium containing antibiotics was replaced with 100 µL of antibiotics-free medium per well. Transfection was executed using the Lipofectamine 2000 transfection reagent (Invitrogen) at a 2:1 ratio (2 μL of Lipofectamine 2000 per 1 μg of nucleic acid) according to the manufacturer’s protocol. HeLa cells were transfected with oligonucleotides **6** and **7** containing the metallacarborane group at concentrations ranging from 1–200 nM and dissolved in 50 µL OPTI-MEM (Gibco BRL). After 5 h of incubation at 37 °C, the transfection mixture was replaced with 200 µL of fresh medium with antibiotics per well. After 48 h of incubation at 37 °C in a 5% CO_2_ atmosphere, the cells were washed once with PBS (without Mg^2+^ or Ca^2+^) and resuspended in 100 μL of PBS (without Mg^2+^ or Ca^2+^). Then, 1 μL of 20 μM DCF-DA solution in DMSO was added to each well. The plate was incubated for 30 min at 37 °C in the dark. The control cells without transfection were treated similarly. After a 15 min of incubation, 1 μL of 1000 nM H_2_O_2_ was added and then cells were incubated for 15 min in the dark. The plate absorbance was measured at two wavelengths, 485 nm and reference 528 nm (plates with black walls and transparent bottoms, Perkin-Elmer). The results are mean values from four independent experiments.

## 4. Conclusions

Therapeutic nucleic acids as gene silencing agents hold enormous promise as future biotherapeutics due to their functional diversity, capacity for specificity and low toxicity. In this study, we proposed a high boron-loaded DNA oligonucleotide modification as potential dual action anticancer agents with antisense/anti-oxidant and BNCT activities. Several important biological features of these metallacarborane-modified DNA oligomers were discovered, including their low cytotoxicity, increased lipophilicity, formation of stable heteroduplexes with complementary RNA strains and function as effective antisense oligonucleotides. Moreover, pro- and anti-oxidant activities were also observed depending on the concentration of the modified oligomer used. The additional value of the metallacarborane modification as an infrared-sensitive nucleic acid label should also be noted. These findings may be of importance for designing therapeutic nucleic acids and facilitate studies on boron clusters as nucleic acid-modifying units.

## Figures and Tables

**Table 1:** Sequences, MALDI-TOF mass-to-charge ratio (*m*/*z*), retention time (R_t_) from the RP-HPLC analysis and maximum wavelength in the ultraviolet–visible (UV) spectrum (λ_max_) for oligonucleotides **6**–**9**, which are modified with metallacarborane cluster nucleoside units (U_B_), oligonucleotides **4** and **5**, which contain 2′-*O*-propargyluridine (U_Pr_) modifications, and non-modified oligomers **1**, **2** and **3**.

No.	Sequence ^3^	*m*/*z* Calc.	*m*/*z* MS MALDI	R*_t_* ^1^ (min)	UV (nm)
**1**	5′-d(TTT CTT TTC CTC CAG AGC CCGA)-3′	6612	6612	16.0	263
**2**	5′-d(AAA GAA AAG GAG GTC TCG GGCT)-3′	6857	6857	15.8	256
**3**	5′-AAA GAA AAG GAG GUC UCG GGCU-3′	7167	7168	15.3	256
**4**	5′-d(TU_Pr_T CU_Pr_T TU_Pr_C CU_Pr_C CAG AGC CCGA)-3′	6772	6775	16.4	263
**5**	5′-d(U_Pr_TU_Pr_CU_Pr_TTU_Pr_C CU_Pr_C CAG AGC CCGA)-3′	6812	6813	16.5	264
**6**	5′-d(TU_B_T CU_B_T TU_B_C CU_B_C CAG AGC CCGA)-3′	8578	8575	22.2	263
**7**	5′-d(U_B_TU_B_ CU_B_T TU_B_C CU_B_C CAG AGC CCGA)-3′	9068	9073	23.9	263
**8** ^2^	5′-d(TTT CTT U_B_TC CTC CAG AGC CCGA)-3′	6881	6884	17.5	263
**9** ^2^	5′-d(TTT CTT U_B_TC CU_B_C CAG AGC CCGA)-3′	7593	7598	20.1	263

^1^ RP-HPLC analysis was performed on a Kinetex 5 µm C18 (250 × 4.6 mm) column. A gradient of buffer B in buffer A was used for elution of the oligonucleotides from the column. Buffer A (0.1 M CH_3_COONH_4_). For oligonucleotides **1**–**5**, buffer B was 40% CH_3_CN in 0.1 M CH_3_COONH_4_. In contrast, 100% CH_3_CN was applied as buffer B for elution of oligonucleotides **6**, **7**, **8** and **9**. The flow rate was 1 mL/min. Detailed gradient data are given in the Materials and Methods section; ^2^ Compounds **8** and **9** were described in a previous report, and the data of their MS, UV and R*t* are published in reference [9]; ^3^ Modified units U_PR_ and U_B_ are highlighted in red.

**Table 2:** Hybridization parameters and melting temperatures of the double strand to single strand duplex transitions for modified oligonucleotides **6** and **7** and non-modified control **1** with complementary DNA or RNA strands **2** or **3**, respectively. The results are mean values from three independent experiments.

Duplex	ΔH [kcal/mol]	ΔS [cal/Kmol]	ΔG [kcal/mol]	*T*_m_ [°C]	*ΔT*_m_ [°C]
**6/2**	−70.3 ± 4.0	−192.0 ± 12.2	−10.7 ± 0.2	49.5 ± 0.2	−16.0
**7/2**	−52.9 ± 1.0	−136.1 ± 2.8	−10.5 ± 0.1	51.9 ± 0.3	−13.6
**1/2**	−120.6 ± 8.6	−330.1 ± 24.9	−18.2 ± 0.9	65.5 ± 0.5	−
**6/3**	−91.9 ± 1.3	−249.3 ± 3.5	−14.6 ± 0.2	60.9 ± 0.5	−8.5
**7/3**	−95.3 ± 7.0	−259.8 ± 20.9	−14.8 ± 0.5	60.4 ± 0.1	−9.0
**1/3**	−107.6 ± 6.8	−288.2 ± 20.2	−18.2 ± 0.6	69.4 ± 0.3	−

## Supplementary Materials

Supplementary Materials are available online, Figure S1: RP-HPLC profiles of oligonucleotides **4** (**A**) and **5** (**B**), which contain 2′-*O*-propargyluridine (U_Pr_ ) and oligonucleotides **6** (**C**) and **7** (**D**), which are modified with metallacarborane cluster nucleoside units (U_B_); Figure S2: Infrared spectra of the oligonucleotides **4** (**A**) and **5** (**B**), and enlarged spectra of **6, 7, 11** and **12** within the B-H diagnostic signals (2200–2800 cm^−1^ region) (**C**); Figure S3: Concentration-dependent silencing activities of control oligonucleotide **13** (5′-d(ATGAAGGTTCAATCTGATTTT) (1–200 nM), metallacarborane 11 and their mixture (**13** + **11**), as determined by a pEGFP-EGFR/RFP dual fluorescence assay in HeLa cells; Figure S4**.** Analysis of ROS generation in HeLa cells by oligonucleotides **1** and **13**, and by ferrocene and oligonucleotide **1**.
