# Supplementary material for: High Boron-loaded DNA-Oligomers as Potential Boron Neutron Capture Therapy and Antisense Oligonucleotide Dual-Action Anticancer Agents ‡
Source: Molecules. 2017 Aug 23;22(9):1393. doi: 10.3390/molecules22091393 (PMC6151803; doi:10.3390/molecules22091393)
Supplement: Supplementary file 1 [file molecules-22-01393-s001.pdf]

molecules

Article Supplementary Materials

High boron-loaded DNA-oligomers as a potential boron neutron capture therapy and antisense oligonucleotide dual-action anticancer agents

Damian Kaniowski[1], Katarzyna Ebenryter-Olbińska[1], Milena Sobczak[1], Błażej Wojtczak[2#], Sławomir Janczak[2], Zbigniew J. Leśnikowski[2*] and Barbara Nawrot[1,*]

[1] Centre of Molecular and Macromolecular Studies, Polish Academy of Sciences, Department of Bioorganic Chemistry, Sienkiewicza 112, 90-363 Lodz, Poland.
[2] Institute of Medical Biology of the Polish Academy of Sciences, Laboratory of Molecular Virology and Biological Chemistry, 106 Lodowa St., 92-232 Lodz, Poland.
[1*] Correspondence: bnawrot@cbmm.lodz.pl
[2*] Correspondence: zlesnik@cbm.pan.pl
[#] Present address: University of Warsaw, Centre of New Technologies, S. Banacha 2c, 02-097 Warsaw.

Content of the Supplementary Materials

1. **Figure S1.** RP-HPLC profiles of oligonucleotides **4** (A) and **5** (B), which contain 2′-*O*-propargyluridine (**U**$_{Pr}$) and oligonucleotides **6** (C) and **7** (D), which are modified with metallacarborane cluster nucleoside units (**U**$_B$).
2. **Figure S2.** Infrared spectra of the oligonucleotides **4** (A) and **5** (B), and enlarged spectra of **6**,**7**, **11** and **12** within the B-H diagnostic signals (2200–2800 cm^{-1} region) (C).
3. **Figure S3.** Concentration-dependent silencing activities of control oligonucleotide **13** (5′-d(ATGAAGGTTCAATCTGATTTT) (1-200 nM), metallacarborane **11** and their mixture (**13**+**11**), as determined by a pEGFP-EGFR/RFP dual fluorescence assay in HeLa cells.
4. **Figure S4.** Analysis of ROS generation in HeLa cells by oligonucleotides **1** and **13**, and by ferrocene and oligonucleotide **1**.

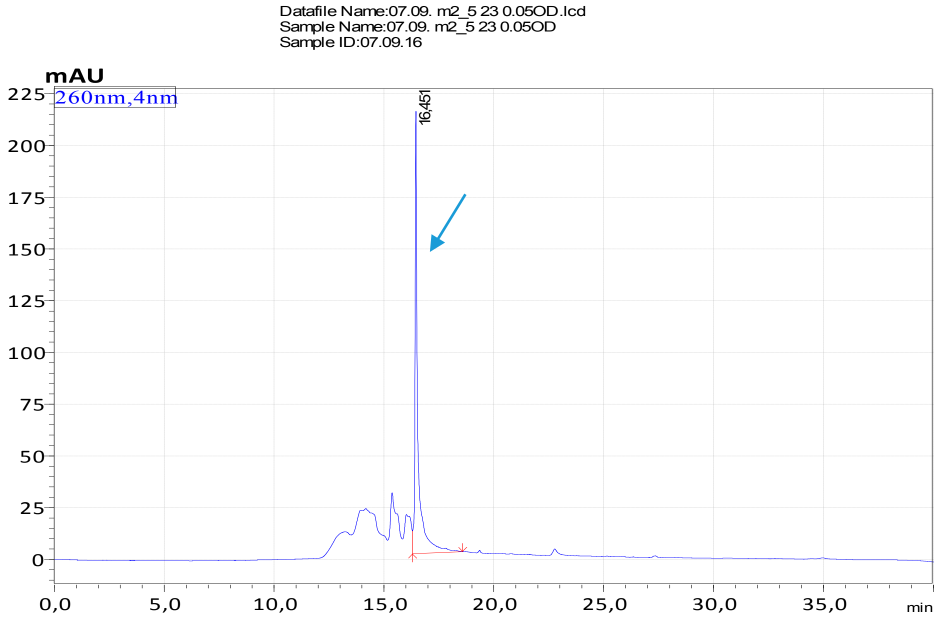
Datafile Name:07.09. m2_5 23 0.05OD.lcd
Sample Name:07.09. m2_5 23 0.05OD
Sample ID:07.09.16
mAU
260nm,4nm
16,451
min

(A)

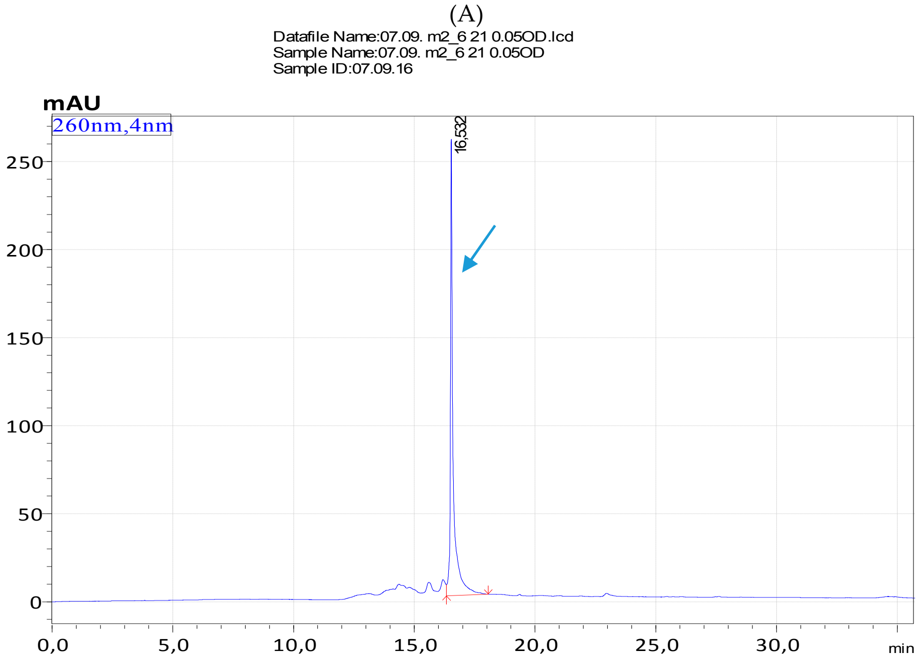
Datafile Name:07.09. m2_6 21 0.05OD.lcd
Sample Name:07.09. m2_6 21 0.05OD
Sample ID:07.09.16
mAU
260nm,4nm
16,532
min

(B)

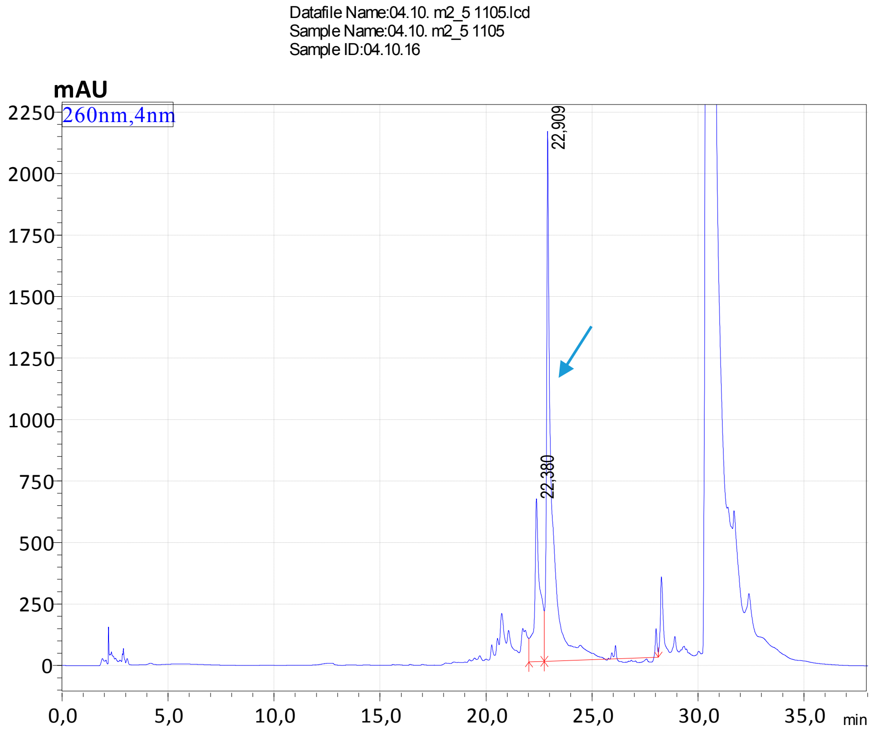

(C)

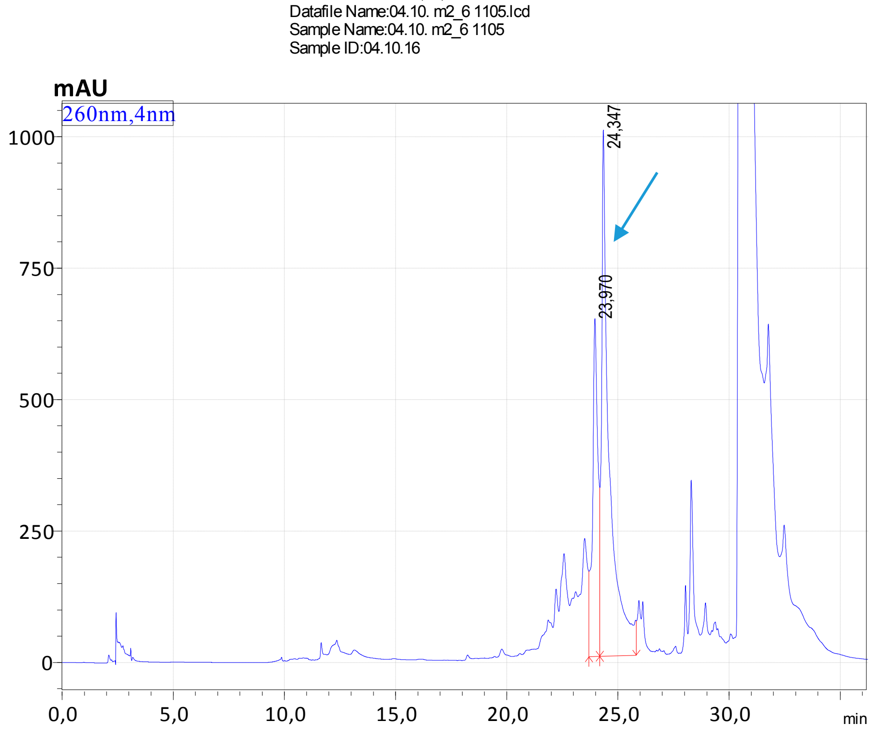

(D)

Figure S1. RP-HPLC profiles of oligonucleotides **4** (A) and **5** (B), which contain 2′-*O*-propargyluridine (**U**$_{Pr}$) and oligonucleotides **6** (C) and **7** (D), which are modified with metallacarborane cluster nucleoside units (**U**$_{B}$). Peaks of the collected compounds are indicated by arrows.

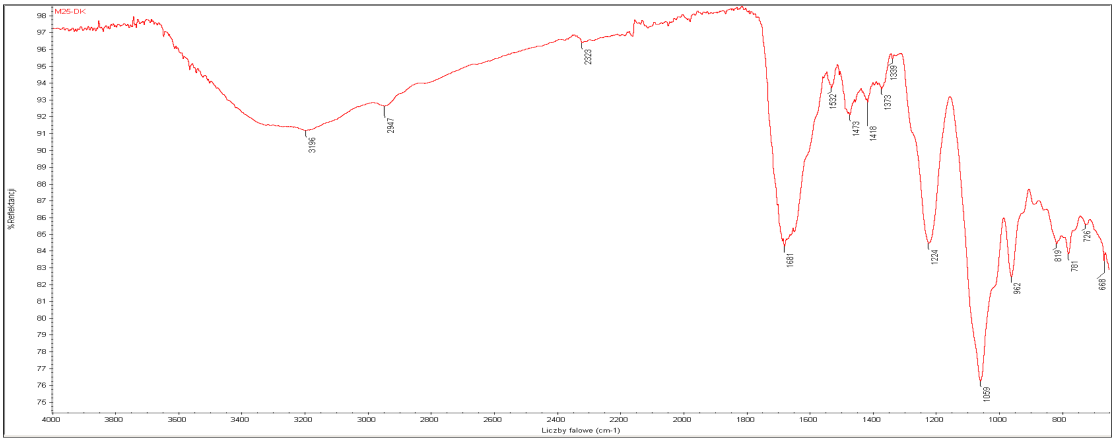
M25-DK
%Reflektancji
Liczby falowe (cm-1)
3196
2947
2323
1681
1532
1473
1418
1373
1339
1224
1059
962
819
781
726
668

(A)

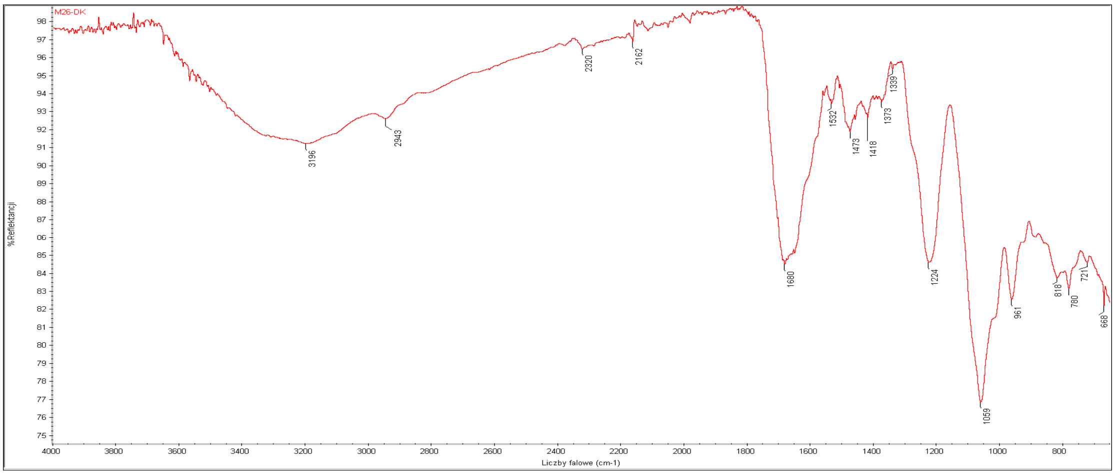
M26-DK
%Reflektancji
Liczby falowe (cm-1)
3196
2943
2320
2162
1680
1532
1473
1418
1373
1339
1224
1059
961
818
780
721
668

(B)

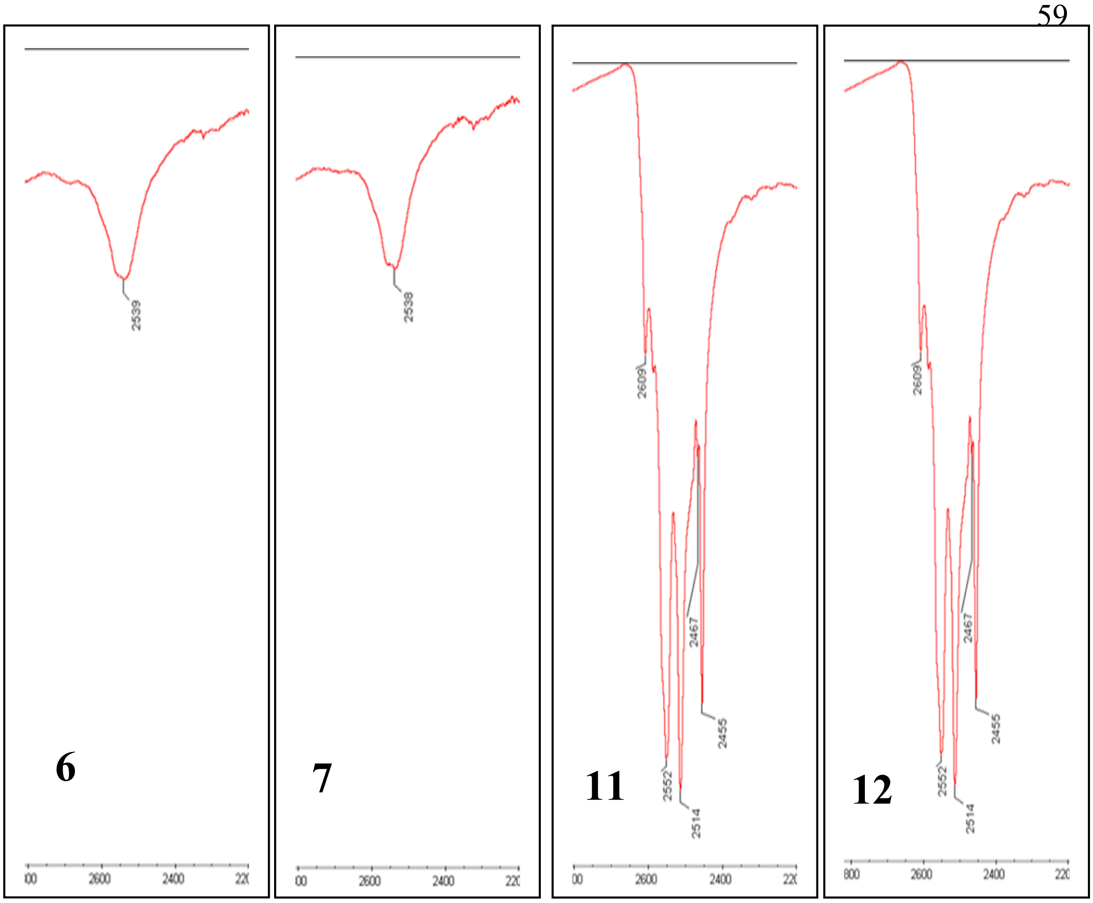

(C)

Figure S2. Infrared spectra of the oligonucleotides **4** (A) and **5** (B), and enlarged spectra of **6**,**7**, **11** and **12** within the B-H diagnostic signals (2200–2800 cm^{-1} region) (C).

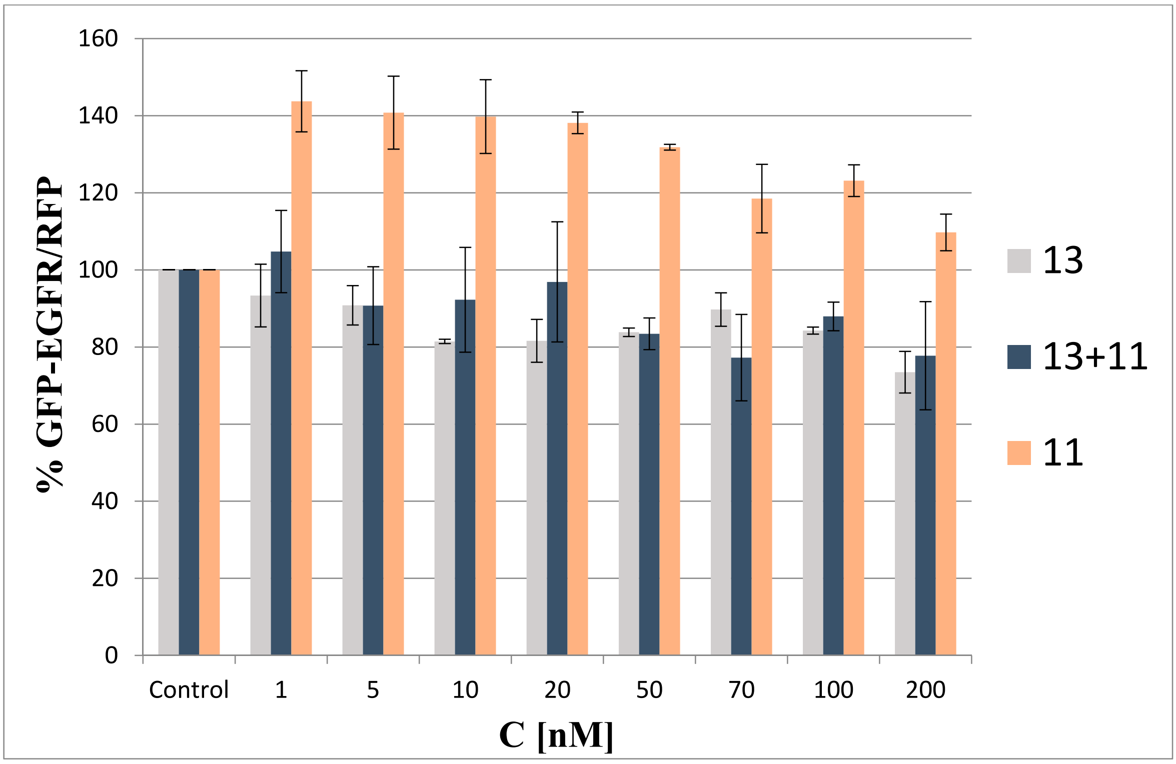

Figure S3. Concentration-dependent silencing activities of control oligonucleotide **13** (5′-d(ATGAAGGTTCAATCTGATTTT) (1-200 nM), metallacarborane **11** and their mixture (**13**+**11**), as determined by a pEGFP-EGFR/RFP dual fluorescence assay in HeLa cells. The cells were transfected with the pEGFP-EGFR and pDsRED-N1 plasmids and then treated (in the presence of Lipofectamine) with oligonucleotide **13** at concentrations ranging from 1-200 nM. Metallacarborane **11** was added to the cells upon oligonucleotide transfection was finished and medium was exchanged. The cells were incubated for the next 48 h. The relative EGFP-EGFR/RFP fluorescence of the cells transfected with the plasmids only was assessed as 100 %. The results are mean values from three independent experiments.

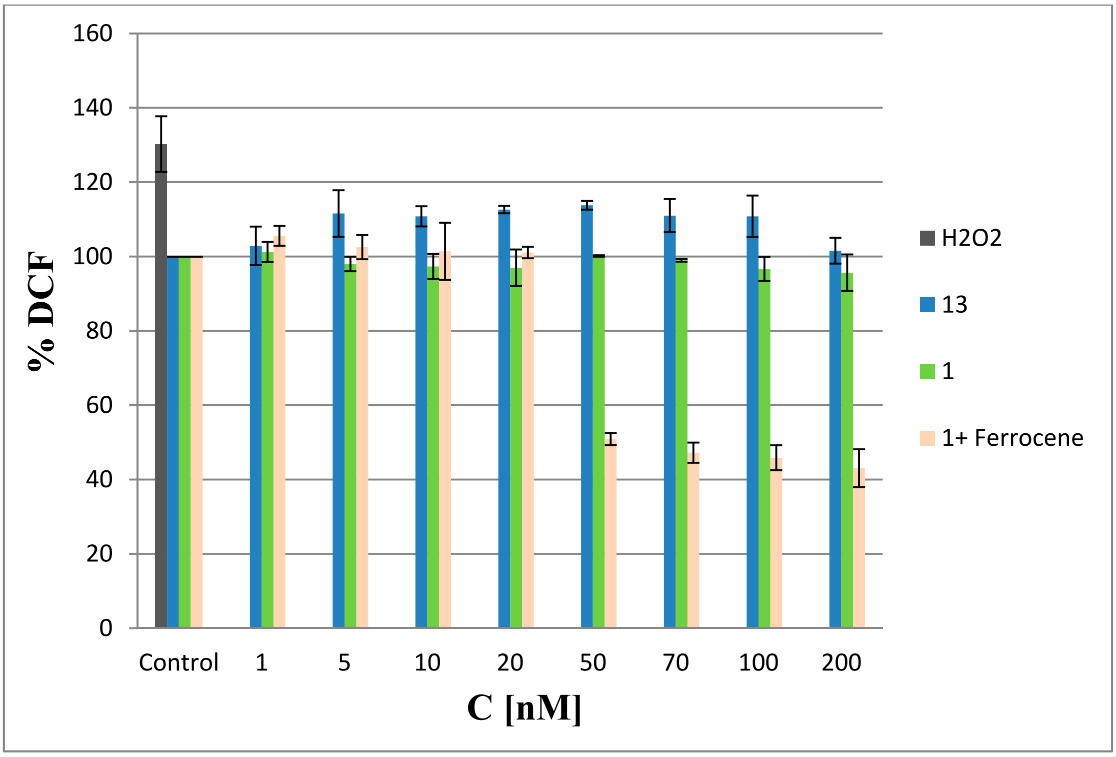

Figure S4. Analysis of ROS generation in HeLa cells by oligonucleotides **1** and **13**, and by ferrocene and oligonucleotide **1**. Cells were transfected with increasing amounts of non-modified oligonucleotide **1** or with control non-active oligonucleotides **13** (5′-d(ATGAAGGTTCAATCTGATTTT) (1-200 nM, 48 h) in the presence of Lipofectamine 2000, or with oligomer **1** in the same concentrations but with addition of ferrocene of 5-fold higher concentration (5-1000 nM). The non-transfected cells and cells treated with 1000 nM H_2O_2 (marked as H_2O_2) were used as controls. The results are mean values from three independent experiments.
